# Local delivery of beta interferon using an adeno-associated virus type 5 effectively inhibits adjuvant arthritis in rats

**DOI:** 10.1099/vir.0.82603-0

**Published:** 2007-06

**Authors:** J. Adriaansen, F. J. Fallaux, C. J. de Cortie, M. J. Vervoordeldonk, P. P. Tak

**Affiliations:** 1 Clinical Immunology and Rheumatology, Academic Medical Center/University of Amsterdam, Amsterdam, The Netherlands; 2 Arthrogen BV, Amsterdam, The Netherlands

## Abstract

Beta interferon (IFN-*β*) is a cytokine with potent immunomodulatory properties and has been described as a promising therapeutic molecule for the treatment of rheumatoid arthritis (RA). IFN-*β* was previously overexpressed intra-articularly using an adenoviral vector in rats with adjuvant arthritis (AA) as a model of RA. This effect was powerful, albeit transient due to the vector chosen. Therefore, in the context of pre-clinical development, a delivery vector optimized for intra-articular gene transfer, recombinant adeno-associated virus type 5 (rAAV5), was selected. To exert an optimal effect, protein production should parallel the course of the disease. For this reason, the gene for IFN-*β* was placed under the control of an inflammation-responsive [nuclear factor (NF)-*κ*B] promoter. After intra-articular injection of the rAAV5 constructs in rats with AA, local transcription of the transgene and production of the IFN-*β* protein was found, leading to a pronounced and sustained effect on paw swelling when the expression was under the control of the NF-*κ*B-responsive promoter. Additionally, a significant beneficial effect was observed on proteoglycan depletion and erosions. Thus, intra-articular overexpression of IFN-*β* using a rAAV5 vector exhibits potential as an innovative therapy for the treatment of RA.

An improved understanding of the pathophysiology of rheumatoid arthritis (RA) has led to the development of several potential new therapeutic approaches based on either cytokines or cytokine inhibitors. Beta interferon (IFN-*β*) is a potent cytokine that exerts a variety of anti-inflammatory and chondroprotective effects in various animal models of RA (Tak *et al.*, 1999; Triantaphyllopoulos *et al.*, 1999; van Holten *et al.*, 2004). In addition, the absence of IFN-*β* was recently shown to exacerbate arthritis in IFN-*β*−/− mice by activation of osteoclasts and stromal cells (Treschow *et al.*, 2005). However, in patients, subcutaneous injections of recombinant IFN-*β* three times a week did not appear to be clinically effective (van Holten *et al.*, 2005). We speculated that the short half-life of IFN-*β* protein *in vivo* and the route of administration may have contributed to insufficiently high levels in the joints for a therapeutic effect.

The feasibility of overexpressing IFN-*β* by intra-articular delivery of gene therapy vectors was therefore investigated in an animal model of RA (Adriaansen *et al.*, 2006a). In this proof of principle study, an adenoviral vector encoding IFN-*β* under the control of the cytomegalovirus (CMV) promoter injected into the joints of arthritic rats was observed to induce a robust therapeutic effect, illustrated by a decrease in paw swelling, a significant shift towards an anti-inflammatory cytokine profile in the synovial tissue and pronounced effects on bone degradation. However, this effect was transient, lasting only 8 days. Adenoviral vector delivery can induce a strong host immune response, leading to elimination of transduced cells (Bessis *et al.*, 2004), while the CMV major immediate-early (IE) promoter can also be silenced *in vivo*, resulting in a rapid decrease of transgene expression (Brooks *et al.*, 2004; van de Loo *et al.*, 2004).

We selected an alternative vector system, derived from adeno-associated virus (AAV) to test in pre-clinical developmental studies. AAV vectors are associated with low immunogenicity, minimal toxicity and are able to transduce both dividing and non-dividing cells (Adriaansen *et al.*, 2006b). Previously, recombinant AAV (rAAV) vectors, packaged in serotype 5 capsids (rAAV5) have been shown to efficiently transduce synovial tissue and they gave rise to stable, long-term intra-articular transgene expression (Adriaansen *et al.*, 2005). In the study presented here, the rat IFN-*β* gene was inserted between the inverted terminal repeats of AAV2 and the DNA was packaged in AAV5 capsids. Both the CMV major IE and a nuclear factor (NF)-*κ*B-responsive promoter were used. Using the latter promoter, expression of IFN-*β* should be disease-inducible, as NF-*κ*B is highly activated in the synovium during inflammation (Tak & Firestein, 2001). We have validated this promoter *in vitro* for its specific NF-*κ*B inducibility (data not shown). In addition, we attempted to boost IFN-*β* production by inclusion of an intron in the expression construct.

Briefly, cDNA encoding the rat IFN-*β* gene (kindly provided by Dr C. Kaynor, Biogen Idec, Cambridge, MA, USA) including its signal peptide was amplified by PCR (Fwd: 5′-CCCGGATCCACCATGGCCAACAGGTGGACCCTCCACATTGCGTTCCTGCTGTGCTTCTCCACCACTGCCCTCCCATCGACTACAAGCAGCTCCAG-3′; Rev: 5′-CCCGGATCCTCAGTTCTGGAAGTTTCTATTAAGTC-3′) and cloned into the *Bam*HI site of the pDC315-shuttle plasmid (Microbix Biosystems). This gene was subsequently inserted between *Nhe*I and *Xho*I sites in the pAAV2-CMV-shuttle plasmid (Applied Viromics). To insert an intron downstream of the rat IFN-*β* gene, pDC315 was digested with *Nhe*I and *Sal*I and ligated into a pCI (Promega) plasmid containing the intron. The gene cassette, including the intron, was subsequently excised with *Sna*BI and *Sal*I and inserted in the pAAV2-CMV-shuttle plasmid. To clone the IFN-*β* gene under the control of the NF-*κ*B-inducible promoter, the CMV promoter was replaced by an NF-*κ*B-responsive promoter. The NF-*κ*B motif was derived from the human immunodeficiency virus long terminal repeat and was cloned into pGmCMV (Promega) using restriction enzymes *Nhe*I and *Xho*I, forming the construct pGNL2. The vector pGNL6 containing six repeats of the NF-*κ*B motif was constructed by digesting pGNL2 with *Nhe*I and *Xho*I and further ligation with the phosphorylated NF-*κ*B oligonucleotides. Thereafter, the NF-*κ*B promoter was digested from pGNL6 by *Nco*I and *Xba*I to replace the CMV promoter in the previously obtained pAAV2-CMV-IFN-*β* plasmid. All constructs were confirmed by sequencing.

rAAV5 vectors were subsequently produced at the UNC Vector Core (Chapel Hill, NC, USA), as described previously (Xiao *et al.*, 1998). Briefly, AAV vectors were generated using the triple transfection protocol and purified using a sequential process of nuclei isolation, density-gradient centrifugation and heparin-sulfate affinity column chromatography. Subsequently, vectors were titrated by dot-blot analysis.

All together, three rAAV5 vectors containing the rat IFN-*β* gene, rAAV5.CMV-IFN-*β*, rAAV5.NF-*κ*B-IFN-*β* and rAAV5.CMV-IFN-*β*-intron were constructed; an empty vector consisting of only the CMV-shuttle vector without the transcription cassette served as a control. Using these vectors, an animal experiment was conducted. Rats (*n*=7 per group) were immunized at the base of their tail with 1 mg *Mycobacterium tuberculosis* H37RA (Difco) in 0.1 ml mineral oil on day 0 (Tak *et al.*, 2001). Paw swelling was usually observed by days 10–11 and measured daily by water displacement plethysmometry. The right ankle joints were injected intra-articularly on day 12 after immunization in animals anaesthetized with isoflurane. The skin was disinfected with ethanol and 5×10^10^ viral molecules (vm) of rAAV5.NF-*κ*B-IFN-*β*, rAAV5.CMV-IFN-*β*, rAAV5.CMV-IFN-*β*-intron or empty vector serving as a control was injected anterolaterally into the right ankle joints in a total volume of 50 μl saline using a 31-gauge needle on a glass syringe (Nguyen *et al.*, 1998). Three weeks later, the animals were sacrificed and hind joints were collected. Hind ankle joints were snap frozen in liquid nitrogen, pulverized using a pestle and mortar, and homogenized in Trizol Reagent (100 mg ml^−1^; Invitrogen) using a tissue homogenizer. For both reverse transcriptase and real-time PCR (*n*=3 per group), total RNA was isolated from the aqueous phase according to the manufacturer’s instructions. RNA was dissolved in DEPC-treated water; the quality was checked by gel electrophoresis and quantified by spectrophotometry. cDNA was synthesized using the iScript cDNA Synthesis kit (Bio-Rad) and 1 μg RNA according to the instruction manual. RT-PCR amplification mixtures (25 μl) contained 25 ng template cDNA, 2× SYBR Green I Supermix (12.5 μl; Bio-Rad) and 300 nM IFN-*β* Fwd (5′-CGTTCCTGCTGTGCTTCTC-3′) and Rev (5′-TGTAACTCTTCTCCATCTGTGAC-3′) primers. Glyceraldehyde-3-phosphate dehydrogenase (GAPDH) was used as an internal reference gene (Fwd: 5′-ATGCCATCACTGCCACTC-3′; Rev: 5′-GGGTAGGAACACGGAAGG-3′). Reactions were run on a MiniOpticon real-time thermal cycler (Bio-Rad). The thermal profile consisted of one cycle at 95 °C for 3 min followed by 40 cycles at 95 °C for 15 s and 59 °C for 45 s. Each assay included (in duplicate): a standard curve of five serial dilutions of IFN-*β* and GAPDH cDNA, a no-template control and 25 ng sample cDNA. Each run was followed by a melting curve. Single control normalization for internal control gene and correction for primer efficiency were calculated as described earlier (Vandesompele *et al.*, 2002).

For immunohistochemical detection of rat IFN-*β* protein in synovial tissue (*n*=4 per group), arthritic paws were fixed in 4 % buffered formalin and decalcified in 15 % EDTA. The paws were then embedded in paraffin and 5 μm sagittal serial sections of the hind ankle joints were cut. Primary IgG antibody (rabbit anti-rat IFN-*β*; Biomedical Laboratories) was incubated overnight at 4 °C, followed by incubation with horseradish peroxidase (HRP)-conjugated swine anti-rabbit immunoglobulin (Dako) for 30 min. For control sections, the primary antibody was omitted. HRP activity was detected using hydrogen peroxide as the substrate and 3-amino-9-ethylcarbazole (Sigma) as the dye. Sections were briefly counterstained with Mayer’s hemalum solution. The signal was subsequently quantified by digital image analysis. Six randomly selected fields within each section were chosen for digitizing the intensity of the signal. These images were acquired on an Olympus microscope, captured using a Charged Coupled Device video camera (Sony) and digitized with a PV100 multimedia 16-bit colour video digitizer card. In the resultant colour images, the area of positive staining and the mean optical density (MOD) were measured by a macro program as described earlier (Kraan *et al.*, 2001). The MOD is proportional to the cellular concentration of protein. The integrated optical density (IOD) is equal to the MOD multiplied by the area of positive staining.

Sections were also stained with Safranin *O*-fast green to determine the loss of proteoglycans. Safranin *O* staining was scored using a semiquantitative scoring system (0–3), where 0 represents no loss of proteoglycans, while a score of 3 indicates complete loss of staining for proteoglycans (Joosten *et al.*, 1999; van Holten *et al.*, 2004). Differences between groups were determined by the Kruskal–Wallis test, followed by a Mann–Whitney U-rank-sum test. *P*<0.05 was considered statistically significant. All analyses were done using spss version 11.5 (SPSS).

Increased levels of rat IFN-*β* mRNA were detected in all animals treated with AAV recombinants. Injection of rAAV5.NF-*κ*B-IFN-*β* resulted in the highest levels of IFN-*β* mRNA, whereas in animals treated with the control vector only low amounts of endogenous IFN-*β* mRNA could be detected by both RT-PCR (Fig. 1a[Fig f1]) and real-time PCR (Fig. 1b[Fig f1]). IFN-*β* protein could clearly be detected by immunohistochemical staining (Fig. 1c[Fig f1]) and was quantified as described above by computer-assisted image analysis. Injection of rAAV5.NF-*κ*B-IFN-*β* resulted in the highest synovial production of IFN-*β*, followed by the rAAV5.CMV-IFN-*β* injected animals. Control and rAAV5.CMV-IFN-*β*-intron treated animals showed only endogenous levels of IFN-*β* (Fig. 1d[Fig f1]).

Interestingly, a pronounced reduction in paw swelling was shown after a single intra-articular injection of rAAV5.NF-*κ*B-IFN-*β*, resulting in sustained amelioration of arthritis until the end of the experiment. In contrast, both rAAV5.CMV-IFN-*β* and rAAV5.CMV-IFN-*β*-intron did not lead to significant improvement in paw thickness compared with control animals (Fig. 2a[Fig f2]). This is most likely because of the lower intra-articular IFN-*β* production compared with the rAAV5.NF-*κ*B-IFN-*β* injected animals, where the promoter is continuously driving expression during inflammation. In addition to the effect on inflammation we investigated the effect on cartilage degradation by Safranin *O* staining (Fig. 2b[Fig f2]), which showed significantly less proteoglycan loss and erosions in treated animals as determined by a semiquantitative scoring system (Joosten *et al.*, 1999; van Holten *et al.*, 2004) (Fig. 2c[Fig f2]). Taken together, we demonstrated that the combined use of a low-immunogenic rAAV5 vector and a disease inducible NF-*κ*B-responsive promoter allows prolonged therapeutic effects in an animal model for RA. Thus, application of IFN-*β* in this context may represent a promising and innovative therapy for the intra-articular treatment of RA.

## Figures and Tables

**Fig. 1. f1:**
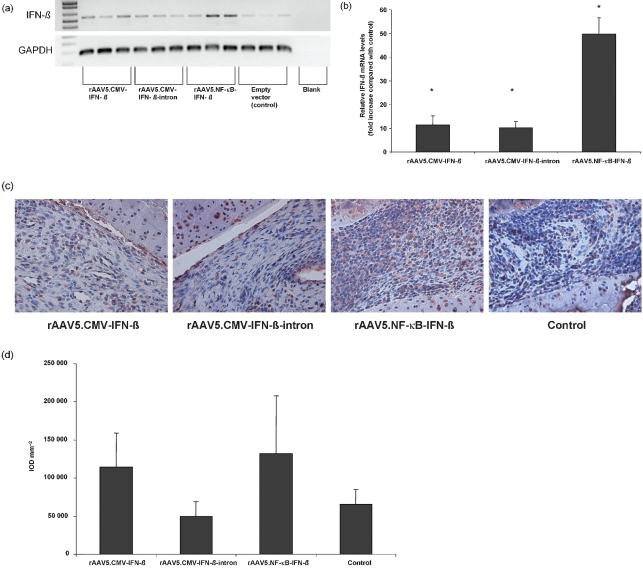
Evaluation of rAAV5-mediated IFN-*β* gene delivery in AA in rats. Relative IFN-*β* mRNA levels in joint tissue. Total RNA was extracted from crushed joints 24 days after intra-articular injection and cDNA was synthesized. RT-PCR (a) and real-time PCR (b) were performed using specific primers for the IFN-*β* gene. C_t_ values were normalized to GAPDH levels and expressed as fold increase compared with matched controls mean±sem. All treated animals significantly overexpressed the transgene in their joints, rAAV5.NF-*κ*B-IFN-*β* gave rise to the highest IFN-*β* mRNA levels (*P<0.05; *n*=3 per group) and intra-articular IFN-*β* protein production after local gene therapy. Rat joints were collected 24 days after injecting the virus, decalcified and embedded in paraffin (*n*=4 per group). Sections were immunohistochemically stained for rat IFN-*β* (c) and quantified by digital image analysis (d). Protein production was highest in rAAV5.NF-*κ*B-IFN-*β* injected animals, followed by the rAAV5.CMV-IFN-*β* group. Control and rAAV5.CMV-IFN-*β*-intron injection did not result in increased IFN-*β* production.

**Fig. 2. f2:**
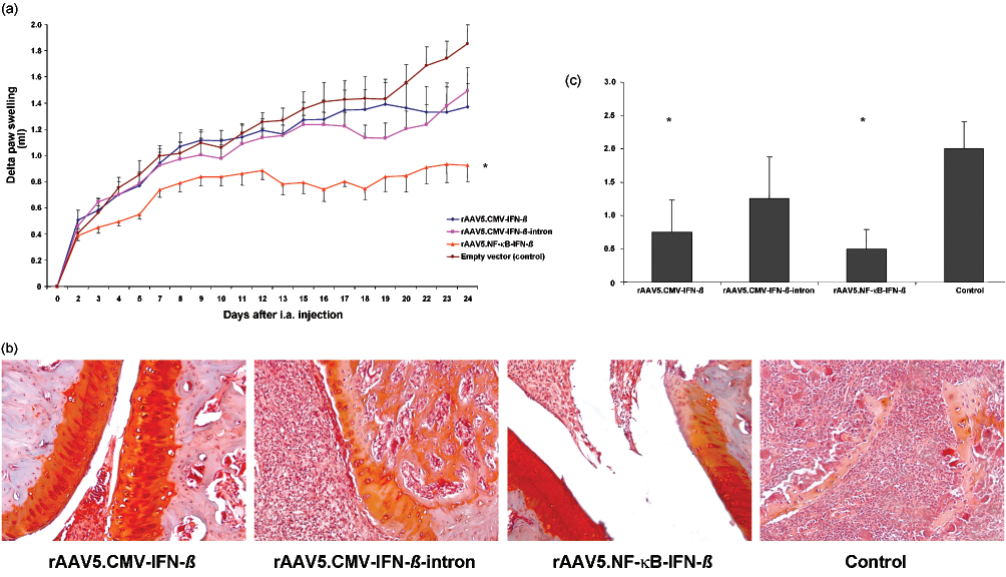
Therapeutic effect of local rAAV5-mediated IFN-*β* gene therapy. Effect of IFN-*β* on paw swelling. To evaluate the effects of IFN-*β* gene therapy on clinical arthritis, seven rats per group were injected intra-articularly (i.a.) into the right ankle joint with the therapeutic vector or empty vector (control) (5×10^10^ vm per joint) on day 12 after arthritis induction. (a) Paw swelling was measured by water displacement plethysmometry. A significant decrease in paw swelling was found in animals treated with the NF-*κ*B driven construct (*P<0.05). (b, c) Cartilage damage in rats with AA treated with local IFN-*β* gene therapy. (b) After 24 days, paws were paraffin embedded and stained with Safranin *O* to detect changes in proteoglycan loss. (c) Stainings were semiquantitatively scored on a scale from 0 to 3, ranging from fully stained cartilage to destained cartilage or complete loss of articular cartilage by two independent observers. A significant reduction in cartilage destruction was seen in animals injected with rAAV5.NF-*κ*B-IFN-*β* and rAAV5.CMV-IFN-*β* compared with the control (*P*<0.05).

## References

[r1] Adriaansen, J., Tas, S. W., Klarenbeek, P. L., Bakker, A. C., Apparailly, F., Firestein, G. S., Jorgensen, C., Vervoordeldonk, M. J. & Tak, P. P. (2005). Enhanced gene transfer to arthritic joints using adeno-associated virus type 5: implications for intra-articular gene therapy. Ann Rheum Dis 64, 1677–1684. 15878906 10.1136/ard.2004.035063 PMC1755308

[r2] Adriaansen, J., Kuhlman, R. R., Holten, J. V., Kaynor, C., Vervoordeldonk, M. J. & Tak, P. P. (2006a). Intraarticular interferon-beta gene therapy ameliorates adjuvant arthritis in rats. Hum Gene Ther 17, 985–996. 16984225 10.1089/hum.2006.17.985

[r3] Adriaansen, J., Vervoordeldonk, M. J. & Tak, P. P. (2006b). Gene therapy as a therapeutic approach for the treatment of rheumatoid arthritis: innovative vectors and therapeutic genes. Rheumatology (Oxford) 45, 656–668. 16510530 10.1093/rheumatology/kel047

[r4] Bessis, N., Garcia Cozar, F. J. & Boissier, M. C. (2004). Immune responses to gene therapy vectors: influence on vector function and effector mechanisms. Gene Ther 11, (Suppl. 1), S10–S17. 15454952 10.1038/sj.gt.3302364

[r5] Brooks, A. R., Harkins, R. N., Wang, P., Qian, H. S., Liu, P. & Rubanyi, G. M. (2004). Transcriptional silencing is associated with extensive methylation of the CMV promoter following adenoviral gene delivery to muscle. J Gene Med 6, 395–404. 15079814 10.1002/jgm.516

[r6] Joosten, L. A., Lubberts, E., Helsen, M. M., Saxne, T., Coenen-de Roo, C. J., Heinegard, D. & van den Berg, W. B. (1999). Protection against cartilage and bone destruction by systemic interleukin-4 treatment in established murine type II collagen-induced arthritis. Arthritis Res 1, 81–91. 11056663 10.1186/ar14 PMC17779

[r7] Kraan, M. C., Smith, M. D., Weedon, H., Ahern, M. J., Breedveld, F. C. & Tak, P. P. (2001). Measurement of cytokine and adhesion molecule expression in synovial tissue by digital image analysis. Ann Rheum Dis 60, 296–298. 11171698 10.1136/ard.60.3.296 PMC1753569

[r8] Nguyen, K. H., Boyle, D. L., McCormack, J. E., Chada, S., Jolly, D. J. & Firestein, G. S. (1998). Direct synovial gene transfer with retroviral vectors in rat adjuvant arthritis. J Rheumatol 25, 1118–1125. 9632074

[r9] Tak, P. P. & Firestein, G. S. (2001). NF-*κ*B: a key role in inflammatory diseases. J Clin Invest 107, 7–11. 11134171 10.1172/JCI11830 PMC198552

[r10] Tak, P. P., Hart, B. A., Kraan, M. C., Jonker, M., Smeets, T. J. & Breedveld, F. C. (1999). The effects of interferon beta treatment on arthritis. Rheumatology (Oxford) 38, 362–369. 10378715 10.1093/rheumatology/38.4.362

[r11] Tak, P. P., Gerlag, D. M., Aupperle, K. R., van de Geest, D. A., Overbeek, M., Bennett, B. L., Boyle, D. L., Manning, A. M. & Firestein, G. S. (2001). Inhibitor of nuclear factor kappaB kinase beta is a key regulator of synovial inflammation. Arthritis Rheum 44, 1897–1907. 11508443 10.1002/1529-0131(200108)44:8<1897::AID-ART328>3.0.CO;2-4

[r12] Treschow, A. P., Teige, I., Nandakumar, K. S., Holmdahl, R. & Issazadeh-Navikas, S. (2005). Stromal cells and osteoclasts are responsible for exacerbated collagen-induced arthritis in interferon-beta-deficient mice. Arthritis Rheum 52, 3739–3748. 16320324 10.1002/art.21496

[r13] Triantaphyllopoulos, K. A., Williams, R. O., Tailor, H. & Chernajovsky, Y. (1999). Amelioration of collagen-induced arthritis and suppression of interferon-gamma, interleukin-12, and tumor necrosis factor alpha production by interferon-beta gene therapy. Arthritis Rheum 42, 90–99. 9920019 10.1002/1529-0131(199901)42:1<90::AID-ANR12>3.0.CO;2-A

[r14] van de Loo, F. A., de Hooge, A. S., Smeets, R. L., Bakker, A. C., Bennink, M. B., Arntz, O. J., Joosten, L. A., van Beuningen, H. M., van der Kraan, P. K. & other authors (2004). An inflammation-inducible adenoviral expression system for local treatment of the arthritic joint. Gene Ther 11, 581–590. 14973543 10.1038/sj.gt.3302182

[r15] Vandesompele, J., De Preter, K., Pattyn, F., Poppe, B., Van Roy, N., De Paepe, A. & Speleman, F. (2002). Accurate normalization of real-time quantitative RT-PCR data by geometric averaging of multiple internal control genes. Genome Biol 3, RESEARCH0034 12184808 10.1186/gb-2002-3-7-research0034 PMC126239

[r16] van Holten, J., Reedquist, K., Sattonet-Roche, P., Smeets, T. J., Plater-Zyberk, C., Vervoordeldonk, M. J. & Tak, P. P. (2004). Treatment with recombinant interferon-*β*reduces inflammation and slows cartilage destruction in the collagen-induced arthritis model of rheumatoid arthritis. Arthritis Res Ther 6, R239–R249. 15142270 10.1186/ar1165 PMC416442

[r17] van Holten, J., Pavelka, K., Vencovsky, J., Stahl, H., Rozman, B., Genovese, M., Kivitz, A. J., Alvaro, J., Nuki, G. & other authors (2005). A multicentre, randomised, double blind, placebo controlled phase II study of subcutaneous interferon beta-1a in the treatment of patients with active rheumatoid arthritis. Ann Rheum Dis 64, 64–69. 15242865 10.1136/ard.2003.020347 PMC1755211

[r18] Xiao, X., Li, J. & Samulski, R. J. (1998). Production of high-titer recombinant adeno-associated virus vectors in the absence of helper adenovirus. J Virol 72, 2224–2232. 9499080 10.1128/jvi.72.3.2224-2232.1998 PMC109519

